# Shift Towards Pro-inflammatory Intestinal Bacteria Aggravates Acute Murine Colitis via Toll-like Receptors 2 and 4

**DOI:** 10.1371/journal.pone.0000662

**Published:** 2007-07-25

**Authors:** Markus M. Heimesaat, André Fischer, Britta Siegmund, Andreas Kupz, Julia Niebergall, David Fuchs, Hannah-Katharina Jahn, Marina Freudenberg, Christoph Loddenkemper, Arvind Batra, Hans-Anton Lehr, Oliver Liesenfeld, Michael Blaut, Ulf B. Göbel, Ralf R. Schumann, Stefan Bereswill

**Affiliations:** 1 Institut für Mikrobiologie und Hygiene, Charité - Universitätsmedizin Berlin, Charité Campus Mitte, Berlin, Germany; 2 Medizinische Klinik I, Charité - Universitätsmedizin Berlin, Campus-Benjamin-Franklin, Berlin, Germany; 3 Max-Planck-Institut für Immunbiologie, Freiburg, Germany; 4 Institut für Pathologie, Charité-Universitätsmedizin Berlin, Campus-Benjamin-Franklin, Berlin, Germany; 5 Institut Universitaire de Pathologie, Centre Universitaire Hospitalier Vaudois, Lausanne, Switzerland; 6 Abteilung Gastrointestinale Mikrobiologie, Deutsches Institut für Ernährungsforschung, Potsdam-Rehbrücke, Nuthetal, Germany; Duke University Medical Center, United States of America

## Abstract

**Background:**

Gut bacteria trigger colitis in animal models and are suspected to aggravate inflammatory bowel diseases. We have recently reported that *Escherichia coli* accumulates in murine ileitis and exacerbates small intestinal inflammation via Toll-like receptor (TLR) signaling.

**Methodology and Principal Findings:**

Because knowledge on shifts in the intestinal microflora during colitis is limited, we performed a global survey of the colon flora of C57BL/10 wild-type (wt), TLR2^-/-^, TLR4^-/-^, and TLR2/4^-/-^ mice treated for seven days with 3.5% dextrane-sulfate-sodium (DSS). As compared to wt animals, TLR2^-/-^, TLR4^-/-^, and TLR2/4^-/-^ mice displayed reduced macroscopic signs of acute colitis and the amelioration of inflammation was associated with reduced IFN-gamma levels in mesenteric lymph nodes, lower amounts of neutrophils, and less FOXP3-positive T-cells in the colon *in situ*. During acute colitis *E. coli* increased in wt and TLR-deficient mice (*P*<0.05), but the final numbers reached were significantly lower in TLR2^-/-^, TLR4^-/-^ and TLR2/4^-/-^ animals, as compared to wt controls (*P*<0.01). Concentrations of *Bacteroides/ Prevotella* spp., and enterococci did not increase during colitis, but their numbers were significantly reduced in the colon of DSS-treated TLR2/4^-/-^ animals (*P*<0.01). Numbers of lactobacilli and clostridia remained unaffected by colitis, irrespective of the TLR-genotype of mice. Culture-independent molecular analyses confirmed the microflora shifts towards enterobacteria during colitis and showed that the gut flora composition was similar in both, healthy wt and TLR-deficient animals.

**Conclusions and Significance:**

DSS-induced colitis is characterized by a shift in the intestinal microflora towards pro-inflammatory Gram-negative bacteria. Bacterial products exacerbate acute inflammation via TLR2- and TLR4-signaling and direct the recruitment of neutrophils and regulatory T-cells to intestinal sites. *E. coli* may serve as a biomarker for colitis severity and DSS-induced barrier damage seems to be a valuable model to further identify bacterial factors involved in maintaining intestinal homeostasis and to test therapeutic interventions based upon anti-TLR strategies.

## Introduction

In inflammatory bowel diseases (IBD) the disturbance of intestinal barrier functions results in increased immunoreactivity against bacterial antigens [1]–[3]. Patients with active intestinal inflammation display accumulation of commensal *Escherichia coli* or *Bacteroides* spp. at inflamed tissue sites [4]–[6]. These bacterial groups, also suspected to trigger intestinal inflammation in acute graft-versus-host-disease after bone marrow transplantation [7], can further potentiate immunopathology by translocation via microlesions and ulcerations [5], [8]. The role of the commensal intestinal microflora in colitis has been studied in a number of experimental models [9], [10], but detailed knowledge on the gut microbiota composition in acute intestinal inflammation is still limited. Recently, we have demonstrated that acute murine ileitis is accompanied by a rigorous *E. coli* overgrowth in the terminal ileum. During acute inflammation, *E. coli* numbers increased by up to nine orders of magnitude and their presence was found to be essential for the induction and progression of ileal immunopathology [8]. High numbers of *E. coli* in the inflamed ileum point towards an important role of bacterial lipopolysaccharide (LPS) in the exacerbation of acute intestinal inflammation. LPS and products from Gram-positive bacteria are recognized by toll-like-receptors (TLRs) 4 and 2, respectively, both of which are expressed in the murine intestinal mucosa [11]. Similar observations have been made in human IBD where TLRs are upregulated at inflamed tissue sites [12], [13]. In line with this, we found that LPS-mediated TLR4 signaling is responsible for a major part of the immunostimulatory potential of *E. coli* in acute ileitis, whereas TLR2, the main receptor for Gram-positive bacteria, was not involved [14].

Acute colitis induced by the barrier-damaging agent dextran-sulfate-sodium (DSS) is exacerbated by gut bacteria, as evidenced by the amelioration of inflammation in germ-free animals and in mice treated with antibiotics [15]. Early studies on roles of bacterial LPS in triggering acute intestinal inflammation revealed that LPS-hyporesponsive C57BL and C3H mice displayed reduced macroscopic signs of colitis as compared to controls and were protected from inflammatory responses initiated by elevated serum LPS levels during inflammation [16]. Another study reported that DSS-induced colitis activity varies with the mouse strain used, but claims that the severity of colitis does not differ in LPS-unresponsive as compared to control mice, irrespective of their genetic background [17]. Furthermore, TLR-mediated sensing of gut bacteria has been suggested to play a role in intestinal homeostasis and TLR4 was shown to limit bacterial translocation during colitis [18]–[21]. In contrast, recent findings demonstrated that TLR-signaling via the adapter protein MyD88 is essential for spontaneous development of colitis in IL10-deficient mice [22]. However, global gut flora analysis in DSS-induced barrier-damage, which could account for some of the contradictory results mentioned above, has not been performed and bacterial species most abundant in acute colitis have not been investigated so far. To further extend our knowledge on potential roles of a bacterial interplay with innate immunity in acute colitis, we performed a global survey of the intestinal microflora and determined numbers of inflammatory cells in DSS-treated C57BL/10 mice lacking TLRs 2 and/or 4. Because major groups of gut bacteria cannot be cultivated, we complemented classical microbiological analyses with culture-independent molecular approaches such as denaturing-gradient-gel-electrophoresis (DGGE) based on separation of PCR-amplified bacterial 16S rRNA gene fragments [8], [14]. Besides identification of gut residents specifically associated with the severity of DSS-induced colitis, we also studied the impact of TLRs 2 and 4 on i) macroscopic signs of acute colitis, ii) changes in relevant immune cell populations in the inflamed colon, and iii) the composition and dynamics of the intestinal microflora in healthy and diseased TLR-deficient animals.

## Results

### Severity of acute intestinal inflammation depends on TLR2- and TLR4-signaling

**Figure 1:**
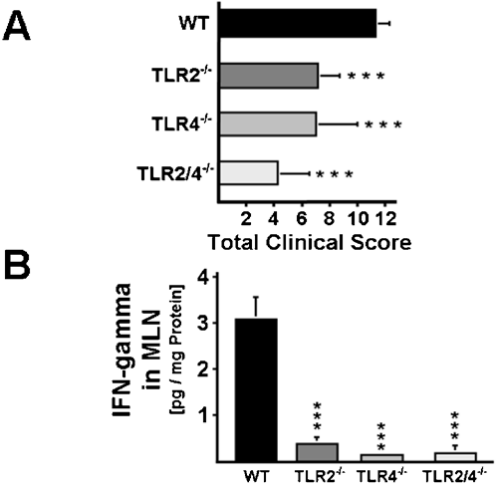
Colitis severity and inflammatory responses in TLR2^-/-^, TLR4^-/-^, and TLR2/4^-/-^ mice, as compared to wt controls. (A) Severity of colitis in DSS-treated animals. Clinical outcome of colitis was recorded in wt mice (WT, n = 10) and in mice lacking TLR2 (TLR2^-/-^, n = 10), TLR4 (TLR4^-/-^, n = 12), or both (TLR2/4^-/-^, n = 11). Clinical parameters of colitis were monitored daily (see methods). The data sets from two independent experiments were pooled. Total colitis scores were analyzed at day eight after DSS-treatment for seven days. (**B**) IFN-gamma concentrations in supernatants of colonic MLN cultures from wt and TLR-deficient animals. Supernatants from MLN cultures of wt mice (WT, black bars, n = 3), and animals lacking TLR2 (TLR2^-/-^, n = 4), TLR4 (TLR4^-/-^, n = 5), or both (TLR2/4^-/-^, n = 4) with colitis were analyzed at day eight after DSS-treatment for seven days. (**A/B**) Mean values, standard deviations and significance levels (as compared to the wt animals) determined by Student's t-test are indicated (^***^, *P*<0.001).

We have recently demonstrated that commensal *E. coli* increase and exacerbate small intestinal inflammation in C57BL/10 mice via LPS-mediated TLR4-signaling [8], [14]. To determine whether TLR2 or TLR4 may be involved in the aggravation of acute colitis, we compared macroscopic signs of disease in C57BL/10 wild-type (wt) mice and animals lacking TLR2, TLR4, or both (Figure 1). After seven days of DSS-treatment, wt mice developed severe signs of colitis as indicated by a total clinical score of 11.3±1 (Figure 1A). In contrast, TLR2^-/-^, TLR4^-/-^, and TLR2/4^-/-^ animals displayed significantly (*P*<0.001) lower clinical scores of 7.1±1.6, 7.0±3, and 4.2±2.3, respectively, as compared to wt animals, thus indicating that colitis is exacerbated via TLR2- and TLR4-signaling (Figure 1A). Furthermore, reduced macroscopic signs of colitis in TLR2^-/-^, TLR4^-/-^, and TLR2/4^-/- ^were associated with diminished inflammatory activity as demonstrated by lower IFN-gamma concentrations (Figure 1B) in colonic mesenteric lymph nodes.

### TLR2- and TLR4-dependent accumulation of effector immune cells in the inflamed colon

**Figure 2:**
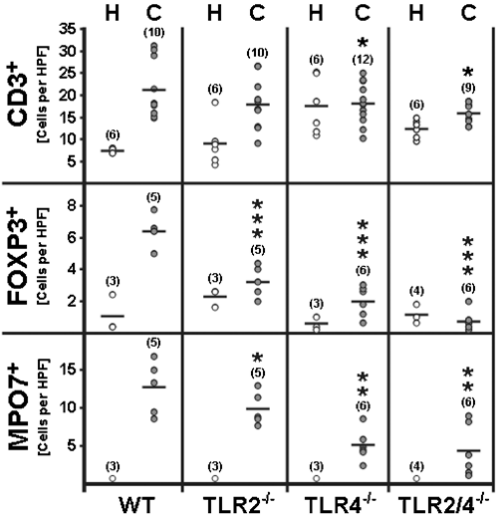
Quantification of immune cells in the colon of DSS-treated mice. Numbers of T-cells, regulatory T-cells, and neutrophils in the colon of DSS-treated wt mice and mice lacking TLR2 and/ or TLR4. The average number of cells positive for CD3, FOXP3, or myeloperoxidase from at least five high power fields (HPF, 400×magnification) per animal were determined microscopically in immunostained colon sections of wt mice (WT), and animals lacking TLR2 (TLR2^-/-^), TLR4^-/-^ (TLR4^-/-^), or both (TLR2/4^-/-^) at day eight after DSS-treatment for seven days (C, grey circles). Healthy animals served as controls (H, white circles). The numbers of analyzed animals are given in parenthesis. Mean values (black bars), and significance levels (as compared to wt animals with colitis) determined by Student's t-test are indicated (^*^, *P*<0.05;^ **^, *P*<0.01; ^***^, *P*<0.001).

The acute stage of DSS-colitis is characterized by recruitment of neutrophils and T-cells to damaged tissue areas [23]. In addition, there is strong evidence that regulatory T-cells accumulate at inflamed tissue sites in DSS-treated mice and in IBD patients [23], [24]. However, their numbers in diseased animals have not been investigated so far. Therefore, we quantified CD3^+^ total T-cells, FOXP3^+^ regulatory T-cells, and myeloperoxidase^+^ neutrophils by immunohistochemistry of colon sections *in situ*. The results demonstrated that numbers of CD3^+^ T-cells were slightly increased in the colons of DSS-treated wt and TLR2-deficient, but not TLR4- and TLR2/4-deficient mice, as compared to the respective naive animals (Figure 2). Most strikingly, the numbers of neutrophils and FOXP3^+^ regulatory T-cells in the inflamed colon were significantly lower in the colons of TLR2^-/-^, TLR4^-/-^, and TLR2/4^-/-^ mice as compared to DSS-treated wt animals (Figure 2). Independent histochemical analyses of HE-stained tissue sections and histological scoring by two pathologists (C.L. and H.A.L.) showed that DSS-induced tissue damage and histopathological changes in the colon were similar in wt, TLR2^-/-^, and TLR4^-/-^ mice, but significantly reduced in TLR2/4^-/-^ mice, as compared to the wt animals (not shown).

### Development of a complex gut flora in TLR2^-/-^ and TLR4^-/-^ mice

**Figure 3:**
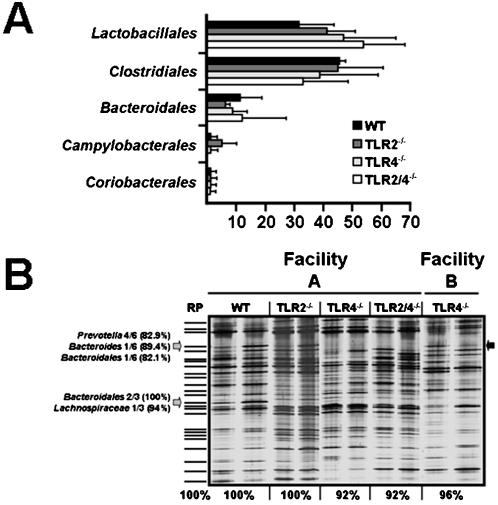
Molecular gut flora analysis in mice lacking TLR2 and/or TLR4. Detailed molecular investigations of the colon microbiota in three months old C57BL/10 mice lacking TLR2, TLR4, or both were performed by analysis of 16S rRNA gene libraries (A) or high-resolution DGGE (B) with primers HDA-1-GC and HDA-2 (see methods). (**A**) Results from gene library analysis. Complete 16S rRNA genes were amplified from total luminal colon content DNA isolated from wt mice (WT, n = 3), and from mice (n = 3 each group) lacking TLR2 (TLR2^-/-^), TLR4 (TLR4^-/-^), or both (TLR2/4^-/-^). The amplicons were cloned and sequenced as described in the Methods section. Bars represent percentage amounts of gut bacterial groups indicated on the Y-axis. (**B**) Results from DGGE analysis of PCR-amplified total bacterial 16S rRNA gene fragments. Each lane shows DGGE profiles of the bacterial flora from the colon of healthy wt mice, and animals lacking TLR2 (TLR2^-/-^), TLR4 (TLR4^-/-^), or both (TLR2/4^-/-^). Mice of identical age (three months) originated from two different animal facilities (as indicated above the lanes). DNA bands, which were absent or weak in DGGE profiles from TLR4^-/-^ and TLR2/4^-/-^ animals are marked by grey arrows. Bacterial species identified by sequence analysis of 16S rRNA gene fragments in the corresponding bands are indicated on the left. Total numbers of DGGE bands in the profile from wt animals were counted and set to 100% in the reference pattern (RP). Presence and absence of bands in profiles from the respective TLR-deficient animals was recorded and similarity values were calculated and expressed as % similarity to the wt RP. The DGGE profiles are representative for at least three mice per group and experiment. Results were reproduced in two independent experiments.

To determine whether sensing of gut bacteria via TLR4 or TLR2 has a general impact on the composition of the intestinal microflora (which could influence the outcome of colitis), we performed a global molecular survey of the colon microbiota in healthy C57BL/10 mice lacking TLR2, TLR4, or both receptors (Figure 3). Therefore, offspring mice were sacrificed, and the bacterial communities within the colon characterized by analysis of cloned bacterial 16S rRNA genes in DNA libraries (Figure 3A), as well as by high-resolution DGGE (Figure 3B). Comparative analysis of 16S rRNA gene libraries revealed that the overall diversity and composition of the gut microflora did not differ significantly among healthy TLR2^-/-^, TLR4^-/-^, and TLR2/4^-/-^ mice, as compared to wt animals (Figure 3A). The number and position of bands in DGGE profiles showed that the molecular pattern of the colon flora of TLR2^-/-^, TLR4^-/-^, and TLR2/4^-/-^ mice were similar to the reference pattern of wt animals, showing 100%, 92%, and 92% concordance, respectively. Two DGGE bands that were less intense in mice lacking TLR4 (Figure 3B, grey arrows) when compared to wt or TLR2^-/-^ animals contained DNA from so far uncultured bacteria of the *Bacteroidales* group (Figure 3B). The fact that i) bacteria represented by the upper band completely absent in TLR4^-/-^ mice were present in the luminal colon contents from TLR4^-/-^ animals that grew up in another facility (Figure 3B, black arrow), and ii) that the lower band was detected in one of the TLR4^-/-^ animals (Figure 3B) provided strong evidence that these differences are most likely caused by interindividual variability.

### Characterization of gut flora changes in acute colitis

**Figure 4:**
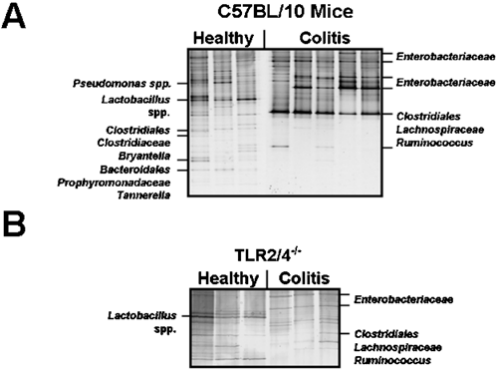
Molecular analysis of predominant bacterial communities in the colon of healthy and diseased mice. (**A**) DGGE profiles of the colon flora in healthy and diseased wt mice. Results are shown for C57BL/10 wt mice without DSS-treatment or at day eight after DSS-treatment for seven days (as indicated above the lanes). Bacterial species were identified by comparative sequence analyses of 16S rRNA gene fragments amplified from DNA eluted from respective DNA bands. The DGGE profiles are representative for at least three mice per group and experiment. (**B**) DGGE profiles of the colon flora in healthy and diseased TLR2/4^-/- ^mice. DGGE profiles of luminal intestinal bacteria from wt and TLR2/4^-/-^ mice were generated by PCR-based analysis of total DNA from colon contents of mice with or without colitis at day eight after DSS-treatment for seven days (as indicated). Bacterial species were identified by comparative sequence analysis of 16S rRNA gene fragments amplified from DNA eluted from respective DNA bands. The DGGE profiles are representative for at least three mice per group and experiment. (**A/B**) Primers GC968F and R1378 (Heimesaat et al., 2006) were used for amplification. Results were reproduced in at least two independent experiments.

**Figure 5:**
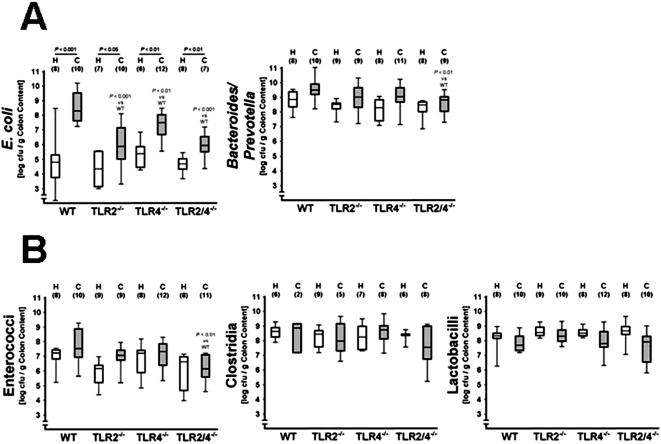
Cultural analysis of gut flora changes in healthy and diseased TLR2^-/-^, TLR4^-/-^, and TLR2/4^-/-^ mice, as compared to wt controls. Bacterial counts of (A) Gram-negative (*E. coli, Bacteroides/ Prevotella* spp.) and (B) Gram-positive bacteria (*Enterococcus* spp., clostridia and lactobacilli) in luminal colon contents from healthy animals (H) and from mice with colitis at day eight after DSS-treatment for seven days (C) as determined by culture. Bacterial species were identified by biochemical analysis and reconfirmed by comparative sequence analyses of 16S rRNA genes. Results are shown for wt mice (WT, above) and for mice lacking TLR2 (TLR2^-/-^), TLR4 (TLR4^-/-^), or both (TLR2/4^-/-^) as indicated on the x-axis. Animal numbers analyzed are given in parentheses. The data sets were pooled from two independent experiments. The boxes represent 75th % and 25th % percentiles of medians (indicated by black bars within the boxes. Maximum and minimum values are indicated by lines above and below boxes, respectively. Significance levels were determined by Mann-Whitney-U-Test. Underlined *P* values shown in black were calculated from comparisons of healthy versus diseased animals. *P* values shown in grey were calculated from the bacterial number in diseased animals (all versus WT).

In order to characterize potential gut flora shifts during DSS-induced barrier damage and to identify gut residents abundant in colitis, we performed a global survey of the gut flora in C57BL/10 mice with or without colitis (Figures 4A and 5). Molecular monitoring of the intestinal bacterial communities by DGGE (Figure 4A) demonstrated that the acute stage of colitis was characterized by a shift towards members of the *Enterobacteriaceae* (Figure 4A). In addition, bacterial communities in the colon from diseased mice were less complex, indicating a loss in bacterial diversity during acute inflammation (Figure 4A). Sequence analysis of bacterial 16S rRNA gene fragments from individual DGGE bands revealed that previously undescribed members of the *Bacteroidales* group, *Bryantella* spp., *Tannerella* spp., clostridia, and lactobacilli disappeared and were not detected in the inflamed colon (Figure 4A). In contrast, a DGGE band containing 16S rRNA genes from bacteria of the *Clostridiales* group was more prominent in samples from the inflamed colon, providing evidence that these bacteria might accumulate during colitis.

A global culture analysis of the gut flora in the inflamed colon of DSS-treated wt mice revealed that concentrations of commensal *E. coli* increased significantly (*P*<0.001) by four orders of magnitude, respectively (Figure 5A). Populations of *Bacteroides/ Prevotella* spp., enterococci, clostridia and lactobacilli were not significantly altered in the inflamed colon (Figures 5A and 5B). Thus, cultural analyses confirmed that the development of colitis is accompanied by shifts in the luminal *E. coli* population.

### Identification of gut flora changes associated with the severity of acute intestinal inflammation

The fact that macroscopic signs and inflammatory parameters of acute intestinal inflammation were reduced in TLR2^-/-^, TLR4^-/-^, and TLR2/4^-/-^ mice prompted us to identify distinct gut bacterial populations associated with colitis severity (Figure 4B and Figure 5). A molecular analysis of the colon microflora composition in DSS-treated TLR2/4^-/-^ animals by DGGE revealed that the intensities of DNA-bands of *Enterobacteriaceae* were weaker when compared to wt animals (compare Figures 4A and B). Furthermore, TLR2/4^-/-^ mice did not display an increased intensity of the DNA band containing a 16S rRNA gene fragment from so far undescribed clostridia (Figure 4B) observed in DSS-treated wt animals (Figure 4A). Most strikingly, a detailed culture-based comparative analysis of the intestinal flora demonstrated that *E. coli* concentrations increased to a lesser degree in DSS-treated TLR2- and TLR4-deficient animals as compared to wt mice (*P*<0.01), indicating that increased colitis severity is associated with higher *E. coli* loads (Figure 5A). In TLR2/4^-/- ^mice, the increase in the *E. coli* numbers was also strongly alleviated, if compared to wt mice (*P*<0.001), and these mice displayed only an approximately 10-fold rise in *E. coli* after colitis induction (Figure 5A). Moreover, DSS-treated TLR2/4^-/-^ mice contained significantly lower luminal loads of *Bacteroides/Prevotella* spp. and enterococci as compared to wt mice (*P*<0.01), whereas all other flora changes in the respective TLR-deficient mice did not significantly differ from those observed in wt controls (Figure 5). Biochemical analyses revealed that the *Bacteroides* population consisted of *B. ovatus*, *B. merdae*, *B. uniformis*, *B. vulgatus,* and *B. thetaiotaomicron*. *Prevotella* spp. were represented by *P. oralis* and *P. buccae*. *Enterococcus* spp. included *E. faecalis*, *E. faecium*, and *E. gallinarum*.

## Discussion

The first global and comprehensive analysis of the colon microflora in the DSS-induced colitis model revealed that acute barrier-damage in the colon is accompanied by accumulation of commensal *E. coli*. Because intestinal overgrowth occurs in different intestinal injuries [25], these flora shifts are most possibly caused by a breakdown of the mucosal physiology. Together with the finding that the lack of TLRs 2 and/or 4 *per se* had no impact on the composition of the intestinal microflora in healthy mice (Figure 3), the lower abundance of *E. coli* in TLR-deficient mice with less macroscopic disease symptoms indicates that *E. coli* can serve as a sensitive biomarker for colitis severity. This is in line with the earlier finding that commensal *E. coli* accumulated drastically during ileitis in our C57BL/10 mice and displayed a strong pro-inflammatory potential to trigger small intestinal inflammation via TLR4 [8], [14]. Similarly, abundant numbers of *E. coli* were associated with inflammation in the colon of IL2^-/-^ mice [26]. Contributions of *E. coli* and other gut bacteria to the aggravation of colitis were shown earlier in mono-associated germ-free IL10^-/-^ and IL2^-/-^ mice [27]-[29] and by the curative effects of antimicrobial therapy [15], [30], [31]. Nevertheless, mechanisms by which accumulating *E. coli* may modulate DSS-induced acute colitis are still not known in detail. The reduced macroscopic signs of colitis displayed by the DSS-treated TLR2^-/-^, TLR4^-/-^, and TLR2/4^-/-^ mice point towards important roles of LPS and TLR2 ligands (such as lipopeptides) in acute inflammatory processes in the colon. In the C57BL/10 mice analyzed here, both ligands seem to potentiate inflammation by TLR-mediated recruitment of neutrophils to inflamed tissue sites. The fact that FOXP3-positive cells were significantly decreased in DSS-treated TLR2^-/-^, TLR4^-/-^, and TLR2/4^-/-^ mice suggests that TLR ligands foster the recruitment and/or proliferation of regulatory T-cells, which can serve as markers for acute intestinal inflammation in this animal model. This is consistent with similar observations in IBD, where inflamed tissue areas contained higher numbers of FOXP3-positive T-cells [24]. Taken together and in line with recent findings [18]-[21], our results underline the important role of gut bacterial sensing by TLRs in maintaining the intricate balance between mucosal immunity and intestinal inflammation.

The results obtained here add important information in as much as bacterial TLR2 ligands may contribute to colitis pathology. In this context it is interesting to note that so far non-cultured Gram-positive bacteria of the *Clostridium* group increased during colitis (as shown by DGGE). Furthermore, the inflamed colon of DSS-treated TLR2/4^-/-^ animals displaying reduced disease symptoms contained significantly lower concentrations of *Bacteroides/Prevotella* spp. and enterococci, as compared to wt-controls. However, since DGGE is not a quantitative technique and because the enterococcus concentrations showed only a tendency to increase during colitis, the potential impact of these observations remains speculative.

In conclusion, DSS-induced colitis is accompanied by a population shift towards *E. coli*, which have the potential to trigger TLR-dependent accumulation of neutrophils and T-cells. This microflora shift towards pro-inflammatory bacteria may help to explain why blockage of TLR-signaling was successfully used to suppress acute intestinal inflammation [32]. Thus, DSS-induced barrier damage seems to be a valuable model to further identify bacterial factors involved in maintaining intestinal homeostasis and to test therapeutic interventions based upon anti-TLR strategies.

## Materials and Methods

### Mice, colitis induction and determination of clinical scores

C57BL/10ScSn wt, TLR2^-/-^, TLR4^-/-^, and TLR2^-/-^/TLR4^-/-^ mice were bred as described [8], [14]. Experiments were conducted according to the German animal protection laws. For colitis induction, mice were treated with 3.5% (wt/vol) DSS (40.000 kDa, MP Biomedicals, Illkirch, France) in drinking water *ad libitum* for seven days. Prior to sacrifice, mice received water without DSS for 24 hours. The intake of the DSS-solution was controlled and mice were weighed daily. Total clinical scores with a maximum of 12 were assessed daily by combined data of weightloss, occurance of blood in stool (Haemoccult™, Beckman Coulter / PCD, Krefeld, Germany), and stool consistence, as described [33].

### Sampling procedures and histologic scoring

Mice were sacrificed with Halothan™ (Eurim-Pharm, Mülheim, Germany) on day eight after induction of colitis. Colon samples were removed under sterile conditions. Histopathology was investigated in paraffin-embedded HE-stained tissue sections. A published standardized histologic score [33] ranging from 0 to 6 was used for blinded evaluation of the inflammatory processes in the colon.

### Immunohistochemistry

For immunostaining, 4 µm sections of formalin-fixed, paraffin-embedded tissue were cut, deparaffinized, and subjected to a heat-induced epitope retrieval step. Slides were rinsed in cool running water, washed in Tris-buffered saline (pH 7.4) before incubation with primary antibodies against CD3 (N1580, Dako, Glostrup, Denmark, dilution 1:10), myeloperoxidase (MPO7, A0398, Dako, 1:10000), and Foxp3 (FJK-16s, eBioscience, 1:100) for 30 min. For detection, biotinylated donkey anti-rat (Dianova, Hamburg, Germany) or rabbit anti-rat (Dako) secondary antibodies were used followed by application of the streptavidinAP kit (K5005, Dako) or the EnVision peroxidase kit (K 4010, Dako). Alkaline phosphatase was revealed by Fast Red as chromogen and Peroxidase was developed with a highly sensitive diaminobenzidine (DAB) chromogenic substrate for approximately 10 minutes. Negative controls were performed by omitting the primary antibody. For each animal, the average number of positive stained cells within at least five independent high power fields (HPF, 400×magnification) were determined microscopically and subjected to statistical analysis as indicated.

### Analysis of the colon microflora

Molecular detection, biochemical identification, and cultural analyses of intestinal bacterial communities were performed as described [8], [14], [34]. Briefly, luminal feces samples were removed for molecular analyses from the distal colon, resuspended in PBS, and centrifuged (16,000×g/10 min/4°C). Total DNA, isolated by phenol extraction as described [8], served as template for PCR amplification of bacterial 16S rRNA genes with consensus primers TPU1 (5′-AGAGTTTGATCMTGGC TCAG-3′, nt 8-27 in the *E. coli* 16S rRNA gene) / RTU8 (5′-AAGGAGGTGATCCANCCRCA-3′, nt 1541-1522 in the *E. coli* 16S rRNA gene). Gene libraries of the amplicons were constructed and analyzed as described [34]. For high-resolution DGGE, which yielded the highest numbers of individual bands from a given sample, the variable region V3 in bacterial 16S rRNA genes was amplified from total gut content DNA with GC clamp (underlined) primer HDA-1-GC (5′-GCCCGGGGCGCGCCCCGGGCGGGGCGGGGGC ACGGGGGGACTCCTACGGGAGGCAGCAGT-3′, nt 339-360 in the *E. coli* 16S rRNA gene) and primer HDA-2 (5′- GTATTACCGCGGCTGCTGGCAC-3′, nt 539-518 in the *E. coli* 16S rRNA gene).

### Determination of IFN-gamma concentrations

MLNs were removed and incubated in 24-flat-bottom well culture plates (Nunc, Wiesbaden, Germany) containing 500 µl serum-free RPMI medium supplemented with penicillin/ streptomycin for 18 h at 37°C. IFN-gamma concentrations in supernatants were determined by ELISA as described [8], [14].

### Statistical analysis

Mean values, medians, standard deviations and levels of significance were determined using Student's *t-*test and the Mann-Whitney-U-Test as indicated. Two-sided probability (*P*) values≤0.05 were considered significant. All experiments were repeated at least twice.
